# Visible spectral distribution of shadows explains why blue targets with a high reflectivity at 460 nm are attractive to tsetse flies

**DOI:** 10.1186/1756-3305-6-285

**Published:** 2013-09-28

**Authors:** Dietmar Steverding

**Affiliations:** 1 BioMedical Research Centre, Norwich Medical School, University of East Anglia, Norwich Research Park, Norwich NR4 7TJ, UK

**Keywords:** Tsetse flies, Blue shadows, Colour attractants

## Abstract

Tsetse flies are known to be attracted by blue fabrics with a high reflectivity at 460 nm. As the visible spectral distribution of shadows has a maximum at around 460 nm it is suggested that the response to blue is due to the search of tsetse flies for shadows to seek for potential hosts, resting sites or cover.

## Letter to the editor

Tsetse flies (*Glossina* spp.) are responsible for the transmission of trypanosomes causing sleeping sickness in humans and nagana disease in cattle. These diseases are restricted to the distribution area of tsetse flies, which are exclusively found in Africa between the Sahara (14° North latitude) and the Kalahari (20° South latitude) [1]. Sleeping sickness is a major cause of morbidity and mortality in humans while nagana disease impairs livestock breeding in many parts of Africa.

One measure of controlling the transmission of trypanosomes is by trapping tsetse flies [2,3]. This control method was already introduced in 1910 [4]. The traps are different in size and shape (vertically oriented biconical constructions and horizontally oriented screens) and consist usually of black and blue panels of cotton cloth [2,3,5]. For tsetse flies of the Palpalis group (*G. palpalis palpalis* and *G. tachinoides*) and the Mortisans group (*G. morsitans morsitans*, *G. pallidipes* and *G. fuscipes*), which include the main vectors for trypanosomes causing sleeping sickness and nagana disease, respectively, it was found that blue fabrics with a high reflectivity at 460 nm and little UV and green-yellow reflectivity are the most effective trap material [2,5-8]. However, it is still a mystery why certain blue fabrics are highly attractive for tsetse flies.

Previously it was suggested that in searching for a resting place tsetse flies are guided by the blueness and darkness of daytime shadows which are tinted bluish by scattered skylight [9]. More recently it was proposed that tsetse flies approach traps when they are in a host- and/or mate-seeking mode [8]. However, hosts themselves create shadows, particularly on the underside of their body. Tsetse flies may respond to these shadows and may be directed by them when seeking for a potential host. In fact, studies have shown that *G. pallidipes* and *G. morsitans morsitans*, two important vectors of nagana disease, feed largely on the belly and lower legs of cattle, parts of the body which are usually shaded [10,11]. Another possibility could be that tsetse flies are directed by blue shadows when searching for cover. However, these hypotheses do not provide an explanation of why blue fabrics with a high reflectivity at 460 nm are so appealing to tsetse flies. The answer to this phenomenon lies in the visible spectral distribution of shadows.

**Figure 1:**
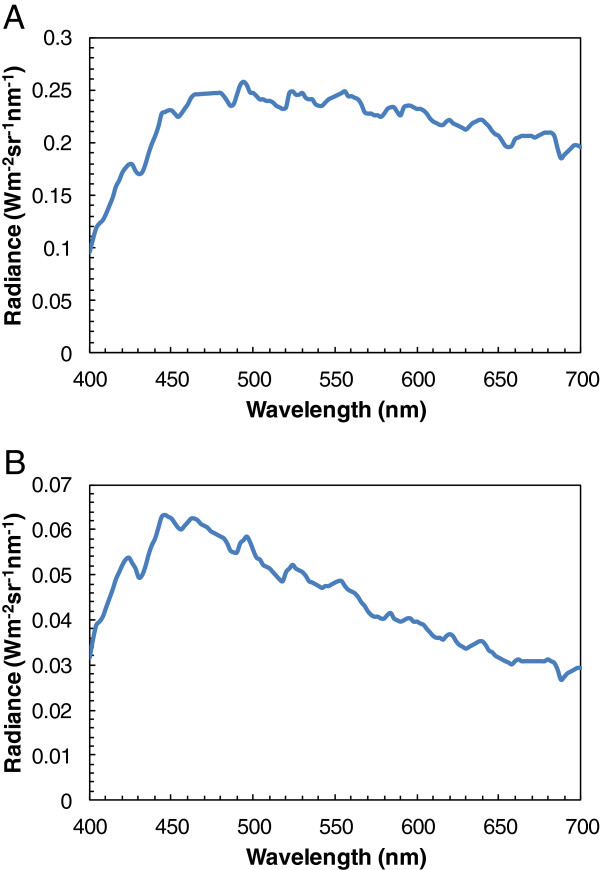
**Visible spectral distribution of sunlight and shadow.** Shown is the radiance versus wavelength for unshaded **(A)** and shaded **(B)** photographic white cards. Data were taken from [12] and reproduced with permission.

The visible spectrum of sunlight is almost evenly spread between 440 to 700 nm (Figure 1A) [12]. In contrast, the visible spectrum of shadows is shifted towards shorter wavelengths (Figure 1B) [12]. Crucially, the visible spectral distribution of shadows has a maximum at around 460 nm (446–464 nm) (Figure 1B) [12]. This characteristic of shadows may explain why tsetse flies are strongly attracted by blue fabrics with a high reflectivity at 460 nm. In this context it is noteworthy to mention that one of the peak sensitivities of the eye of tsetse flies is also at 460 nm [13,14].

## Conclusion

In conclusion, it is suggested that in search for a resting place, cover, and/or a host, tsetse flies are guided by the maximum, at around 460 nm, of the visible spectrum of shadows. Blue fabrics with a high reflectivity at 460 nm are probably mistaken by tsetse flies as shadows, which explains their response to these blue dyed cloths. Whether this finding can be utilised in the development of better performing traps by imitating shadows, remains to be shown. For example, the development of 460 nm emitting “insectocuters” may be possible; these could be powered by batteries and placed near livestock sheds. In addition, in the present digital age, cameras, mobile phone apps etc. could be developed to capture spectral photographic images at around 460 nm to create a “tsetse-eye” view. This might help in identifying sites attractive to tsetse flies on either animals or vegetation/environment that could assist to guide spraying or uprooting.

## Competing interests

The author declares that he has no competing interests.
